# Characteristics and Differences in the Antler Velvet Microbiota During Regeneration

**DOI:** 10.3390/microorganisms13010036

**Published:** 2024-12-27

**Authors:** Yongxiang Li, Yuhang Zhu, Bo Yang, Shaochen Yu, Songze Li, André-Denis G. Wright, Rui Du, Huazhe Si, Zhipeng Li

**Affiliations:** 1 Joint International Research Laboratory of Modern Agricultural Technology, Ministry of Education, Jilin Agricultural University, Changchun 130118, China; 2 College of Animal Science and Technology, Jilin Agricultural University, Changchun 130118, China; 3 School of Biological Sciences, University of Oklahoma, Norman, OK 73019, USA; 4 Jilin Provincial Engineering Research Center for Efficient Breeding and Product Development of Sika Deer, Jilin Agricultural University, Changchun 130118, China; 5 Key Laboratory of Animal Production, Product Quality and Security, Ministry of Education, Jilin Agricultural University, Changchun 130118, China

**Keywords:** antler velvet, microbiota, wound healing, *Staphylococcus*, *Corynebacterium*

## Abstract

The skin surface has a complex and dynamic ecosystem inhabited by a diverse microbiota. The wound formed by antler velvet shedding can naturally achieve regenerative restoration, but the changes in microbial composition that occur during antler velvet regeneration are largely unknown. In this study, we analyzed the antler velvet microbiota of sika deer at 15 days (Half) and 30 days (Full) post-pedicle casting using 16S rRNA gene sequencing. A total of 2659 OTUs were identified, which were assigned to 26 phyla, 304 families, and 684 genera. The core microbiota of the two groups were mainly composed of *Atopostipes* spp., *Corynebacterium* spp., *Burkholderia* spp., *Staphylococcus* spp., and *Paracoccus* spp. In comparison with the Full group, the Shannon, Simpson, Ace, and Chao 1 indices were significantly decreased in the Half group (*p* < 0.05). Principal coordinate analysis showed that there were significant differences in the microbial community between the Half and Full groups based on Bray–Curtis dissimilarity, weighted Unifrac distance, and unweighted Unifrac distance (*p* < 0.05). The relative abundances of bacteria belonging to the genera *Staphylococcus*, *Romboutsia*_B, and *Dietzia* increased significantly in the Half group, while the abundances of bacteria belonging to the genera *Atopostipes*, *Psychrobacter*, and *Faecousia* increased significantly in the Full group (*p* < 0.05). Correlation analysis showed that the relative abundances of bacteria belonging to the genera *Staphylococcus*, *Romboutsia*_B, and *Dietzia* positively correlated with arginine and proline metabolism (*p* < 0.05). These findings demonstrate that antler velvet regeneration is accompanied by distinct changes in microbial composition and highlight the potential roles of key taxonomy in wound healing and tissue regeneration.

## 1. Introduction

The skin is the largest and most external organ of mammals, with its structural integrity acting as a vital barrier against pathogen invasion [1]. However, millions of people annually suffer skin injuries caused by trauma, surgery, or burns [2]. The common outcome of adult mammalian wound healing is that the injured dermis is replaced by a fibrotic scar that impairs tissue function and leads to significant undesirable psychological and physical distress [3,4,5]. In addition, wounds may also become chronic, with the healing process lasting for months, significantly impacting quality of life [6]. Therefore, effective treatments to promote wound healing and reduce scarring in humans are urgently needed. Cervids are a unique kind of mammal due to their growth of antlers [7]. The particular importance of deer antlers lies in the fact that they are completely covered by a layer of hair-bearing skin, called velvet, throughout their growth, and until they have attained maturity. Deer antler velvet is also richly embedded with hair follicles and sebaceous glands [8]. Moreover, the transplantation of antler stem cells into radiation-induced cutaneous wounds has been shown to shorten wound healing time and reduce scar formation in rats [9]. It also has been demonstrated that antler stem cell-derived exosomes enhance both the speed and quality of full-thickness skin wound healing in rats [10]. In addition, injection of antler velvet polypeptides enhances the ability of adipose-derived stem cells to promote wound healing in mice with chronic skin ulcers [11]. Hence, antler velvet represents a unique model to examine adult skin regeneration.

To understand the molecular events enabling antler velvet regeneration, Sinha et al. (2022) demonstrated that velvet fibroblasts adopt an immunosuppressive phenotype that restrict leukocyte communication and hastens immune resolution to promote scar-free regeneration in the velvet of reindeer [8], indicating the important role for the immune response in skin regeneration. However, the skin also represents a highly complex and dynamic ecosystem colonized by trillions of microbes [12], due to the micro-environment of the surface, which is desiccated, aerobic, saline, and acidic, and the invaginations are relatively anaerobic and lipid-rich [13]. Interestingly, recent findings suggested that the skin microbiota also plays a pivotal role in maintaining skin barrier integrity and modulating immune responses, particularly during the process of wound healing [14,15]. Although the skin microbiota of adult mammals, such as squirrels, cattle, sheep, pigs, and dogs, is mainly composed of Actinobacteria (*Micrococcus* spp.) and Firmicutes (*Staphylococcus* spp.) the community structure and composition vary by host species [16,17,18,19,20]. We thus hypothesized that antler velvet itself may harbor a distinct microbial community. Of importance, recent studies highlight the beneficial effects of commensal skin bacteria in facilitating wound healing and tissue regeneration. For instance, *Staphylococcus* spp. can induce IL1β signaling and activate keratinocytes to stimulate hair follicle regeneration and accelerate wound healing through IL1R/Myd88 [21]. Additionally, lipoteichoic acid, a product of *Staphylococcus epidermidis*, has been shown to inhibit toll-like receptor 3 (TLR3) signal transduction. This inhibition reduces inflammatory cytokine release by keratinocytes, thereby alleviating injury-induced inflammation and supporting a favorable healing environment [15]. Here, we hypothesized that the microbiota undergoes significant changes during antler velvet regeneration.

In this study, we collected microbial samples from antler velvet during the Half and Full regeneration stages and conducted 16S rRNA gene sequencing to: (1) illustrate the microbial composition and predicted functions of antler velvet microbiota, and (2) reveal dynamic changes in the microbiota during this process. Our results uncovered the potential role of the microbiota in facilitating antler velvet regeneration, offering novel insights into wound healing and skin regeneration.

## 2. Materials and Methods

### 2.1. Animals and Sample Collection

In this study, a total of 15, 4-year-old, male sika deer were used, including 6 sika deer at the Half regeneration stage of antler velvet (Figure S1a, 15 days after pedicles casting, Half group) and 9 sika deer at the Full regeneration stage of antler velvet (Figure S1b, 30 days after pedicles casting, Full group). The microbiota of antler velvet was collected using eSwabs (COPAN e480C, Brescia, Italy) according to previous methods [22,23,24]. In brief, fresh gloves were worn during each sample collection. Samples were collected from both the left and right antlers (1 cm × 4 cm). The swab was premoistened in preservation medium. A flocked swab was swabbed 20 times in a forward and backward motion across the skin to obtain the skin scrapings. After that, each swab tip was cut, placed in a 2.0-mL sterile tube, and frozen in liquid nitrogen, then stored at −80 °C until DNA extraction. All animal procedures were approved by the Animal Ethics Committee of Jilin Agricultural University (No. 20210314002).

### 2.2. DNA Extraction, PCR Amplification, and High-Throughput Sequencing

Genomic DNA was extracted from the velvet swabs using a PowerSoil DNA Isolation Kit (MoBio Laboratories, Carlsbad, CA, USA), following previous methods [23,25]. The frozen swabs were placed in bead tubes, which were incubated at 65 °C for 10 min, followed by vortexing for 1 min at a maximum speed of 6.5 m/s. The remaining steps were carried out according to the manufacturer’s instructions. DNA integrity was confirmed by 1% agarose gel electrophoresis, and DNA concentration was measured with a NanoDrop spectrophotometer (Thermo Fisher Scientific, Waltham, MA, USA). The primers 341F (5′-CCTAYGGGRBGCASCAG-3′) and 806R (5′-GGACTACNNGGGTATCTAAT-3′) [26] were used to amplify the V3-V4 region of the bacterial 16S rRNA gene. Amplification was performed as described by Kennedy et al. (2014) [27] and Ross et al. (2018) [28]. Each amplification was performed in triplicate to minimize potential PCR bias from low biomass samples. Amplicons were purified using a QIAquick PCR Purification Kit (QIAGEN, Valencia, CA, USA) and quantified using a Qubit 2.0 (Invitrogen, Carlsbad, CA, USA). The PhiX Control library (Illumina, San Diego, CA, USA, 20%) was combined with the amplicon library, followed by normalization and sequencing on an Illumina PE MiSeq platform to generate 250 bp paired-end reads.

### 2.3. Bioinformatics Analysis

Paired-end reads were quality controlled used FASTP [29] and assembled into contigs using FLSAH [30], and the resulting sequences were imported into QIIME 2 [31] for further analysis. Based on a sequence similarity level of 97%, the sequences were clustered into operational taxonomic units (OTUs) using UPARSE [32], and then potential chimeric sequences were removed using UCHIME [33]. The representative sequences for each OTU were analyzed by RDP classifier against the SILVA database (version 138) using a confidence threshold of 0.7 [34]. A phylogenetic tree of the representative OTUs was constructed using QIIME 2.

The microeco package [35] was used to calculate alpha diversity, including Shannon, Simpson, Ace, and Chao 1 indices. Principal coordinate analysis (PCoA) was used to visualize variation in the microbial community and structure based on a Bray–Curtis dissimilarity matrix, weighted UniFrac distance, and unweighted UniFrac distance. Group similarity was calculated by permutational multivariate analysis of variance (Adonis). Tax4Fun package was used to predict the functional profiles of the microbiota based on the Kyoto Encyclopedia of Genes and Genomes (KEGG) database [36]. Linear discriminant analysis (LDA) effect size (LEfSe) [37] was employed to identify differences of the relative abundances of KEGG pathways between the Half and Full groups. The psych package was used to calculate Spearman’s correlation coefficient among the microbiota for the Half and Full groups. Significant correlations (*p* < 0.05, |r| > 0.6) were visualized using Gephi [38].

### 2.4. Statistics Analysis

Wilcoxon rank test was used to determine the significance of alpha diversity indices, the relative abundances of different taxonomic units, and KEGG pathways between the Half and Full groups. The *p* values were corrected using the Benjamini–Hochberg method, with a threshold of *p* < 0.05 considered statistically significant.

## 3. Results

### 3.1. Characterization of the Microbial Composition of Antler Velvet

**Figure 1:**
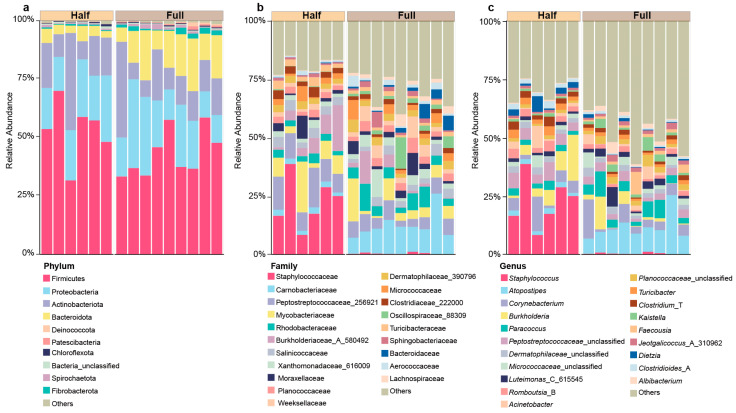
The microbial composition of antler velvet at the (**a**) phylum, (**b**) family, and (**c**) genus levels between the Half and Full groups.

We obtained a total of 556,951 sequences from the 15 samples. After quality control, a total of 543,565 reads were retained and classified into 2659 OTUs. The taxonomy analysis showed that these OTUs were classified into 26 phyla, 304 families, and 684 genera (Table S1). At the phylum level, bacteria belonging to the Firmicutes (Half = 53.27 ± 5.23%, Full = 43.12 ± 3.28%), Proteobacteria (Half = 20.96 ± 2.05%, Full = 21.26 ± 3.21%), Actinobacteriota (Half = 18.59 ± 4.95%, Full = 15.61 ± 3.52%), and Bacteroidota (Half = 4.49 ± 0.63%, Full = 14.99 ± 1.90%) were the dominant microbiota (Figure 1a). At the family level, the following taxa were present: Staphylococcaceae (Half = 22.48 ± 4.36%, Full = 0.43 ± 0.12%), Peptostreptococcaceae_256921 (Half = 11.27 ± 1.47%, Full = 5.84 ± 0.66%), Carnobacteriaceae (Half = 2.19 ± 0.20%, Full = 11.97 ± 1.89%), and Mycobacteriaceae (Half = 8.38 ± 2.88%, Full = 5.73 ± 1.78%) (Figure 1b). At the genus level, the bacteria were dominated by *Staphylococcus* spp. (Half = 22.58 ± 4.36% Full = 0.41 ± 0.12%), *Atopostipes* spp. (Half = 1.94 ± 0.17%, Full = 11.22 ± 1.92%), *Burkholderia* spp. (Half = 8.38 ± 2.88%, Full = 5.73 ± 1.78%), and *Corynebacterium* spp. (Half = 5.56 ± 2.01%, Full = 5.18 ± 1.63%) (Figure 1c).

### 3.2. Comparison of the Microbial Composition Between the Half and Full Groups

**Figure 2:**
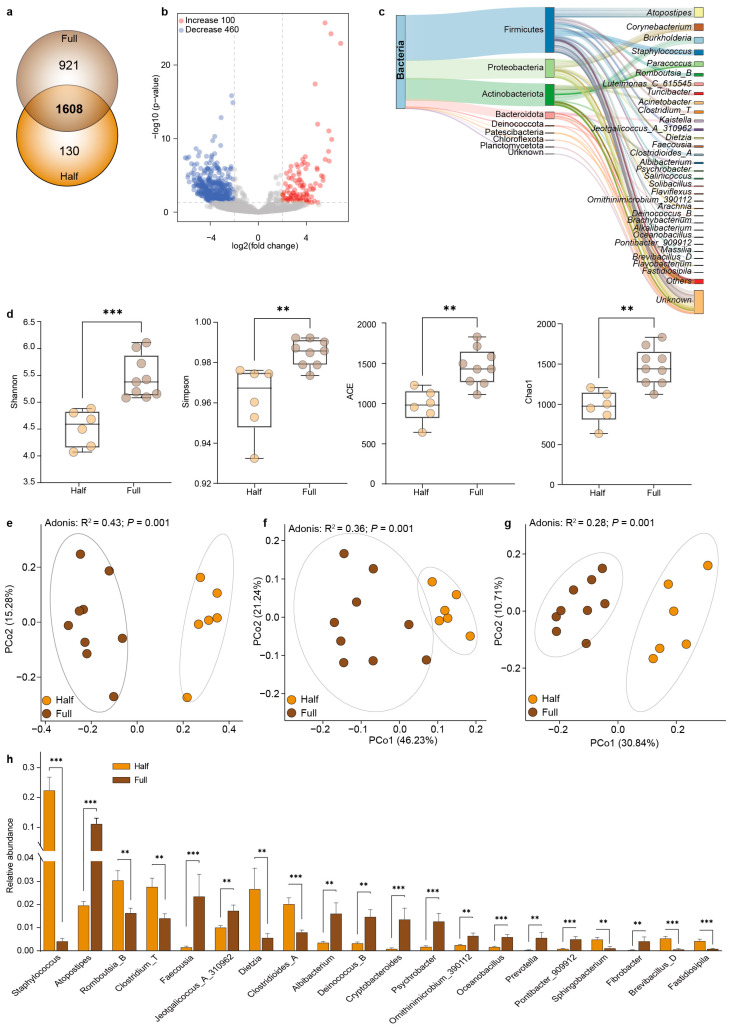
Comparison of microbial community composition between the Half and Full groups. (**a**) Comparison of the number of OTUs between the Half and Full groups. (**b**) Volcano plot showing the significantly changed OTUs between the Half and Full groups. (**c**) Taxonomic classification of the core OTUs. (**d**) Comparison of alpha diversity indices between the Half and Full groups. PCoA revealing differences in the microbial community based on (**e**) Bray–Curtis dissimilarity matrix, (**f**) weighted Unifrac distance, and (**g**) unweighted Unifrac distance. (**h**) Significantly different genera between the Half and Full groups. ** *p* < 0.01, *** *p* < 0.001.

The comparison showed that 1608 OTUs were shared between the Half and Full groups, and a total of 130 and 921 OTUs were unique to the Half group and the Full group, respectively (Figure 2a). The abundances of 100 OTUs and 460 OTUs in the Half group significantly increased and decreased, respectively, in comparison with the Full group (*p* < 0.05) (Figure 2b). Furthermore, we identified a total of 184 core OTUs that were present in all samples (Figure 2c). These 184 OTUs were assigned to eight phyla, such as Firmicutes, Proteobacteria, Actinobacteota, and Bacteroidota, which accounted for more than 97% microbiota. Moreover, these 184 OTUs were classified into 69 genera, such as *Atopostipes* spp., *Corynebacterium* spp., *Burkholderia* spp., *Staphylococcus* spp., and *Paracoccus* spp.

Next, we compared the microbial diversity between the Half and Full groups, and found that the Shannon (*p* < 0.001), Simpson (*p* < 0.01), Ace (*p* < 0.01), and Chao1 (*p* < 0.01) indices in the Half group were significantly lower than those in the Full group (Figure 2d). The PCoA results revealed the clear and significant separation of microbiota between the Half and Full groups (*p* < 0.01) based on the Bray–Curtis dissimilarity matrix (Figure 2e), weighted Unifrac distance (Figure 2f), and unweighted Unifrac distance (Figure 2g). We then identified differences in microbial composition during antler velvet regeneration (Figure 2h). The results showed that the relative abundances of *Staphylococcus* spp., *Romboutsia*_B, *Clostridium*_T, *Dietzia* spp., *Clostridioides*_A, *Sphingobacterium* spp., *Brevibacillus*_D, and *Fastidiosipila* spp. were significantly increased in the Half group (*p* < 0.01) compared with the Full group, while the relative abundances of *Atopostipes* spp., *Faecousia* spp., *Jeotgalicoccus*_A_310962, *Albibacterium* spp., *Deinococcus*_B, *Cryptobacteroides* spp., *Psychrobacter* spp., *Ornithinimicrobium*_390112, *Oceanobacillus* spp., *Prevotella* spp., *Pontibacter*_909912, and *Fibrobacter* spp. were lower (*p* < 0.01).

### 3.3. Co-Occurrence Network Analysis of the Microbial Interactions

**Figure 3:**
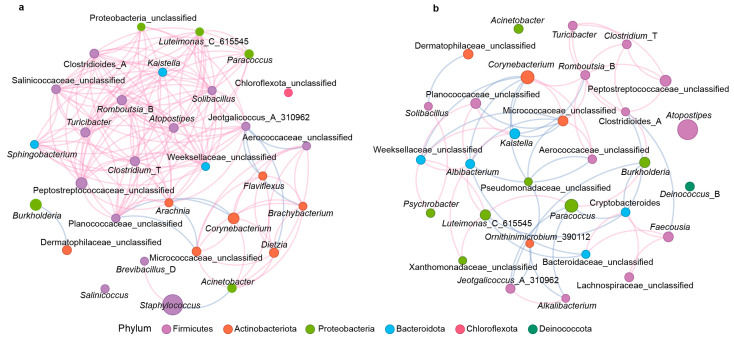
The co-occurrence network of the microbial community at the genus level based on Spearman correlation in the Half (**a**) and Full (**b**) groups (r > 0.6 or r < −0.6, *p* < 0.05). The pink and blue lines indicate positive and negative correlations, respectively.

The co-occurrence network showed that there was a strong positive correlation among microbiota in the Half group (148 edges, positive edges = 138, negative edges = 10; Figure 3a), while the co-occurrence network in the Full group was relatively relaxed (64 edges, positive edges = 37, negative edges = 27; Figure 3b). In the largest positive correlation module, a total of 17 microbial genera, such as *Clostridides*_A, *Clostridium*_T, *Turicibacter*, *Romboutsia*_B, *Sphingobacterium*, *Arachnia*, *Atopostipes*, *Solibacillus*, *Kaistella*, *Paracoccus*, *Luteimonas*_C_615545, and *Jeotgalicoccus*_A_310962, showed significant positive correlations in the Half group, while eight genera such as *Clostridides*_A, *Clostridium*_T, *Corynebacterium*, *Turicibacter*, and *Romboutsia*_B were positively correlated in the Full group.

### 3.4. Changes in the Function of Antler Velvet Microbes Between the Two Groups

**Figure 4:**
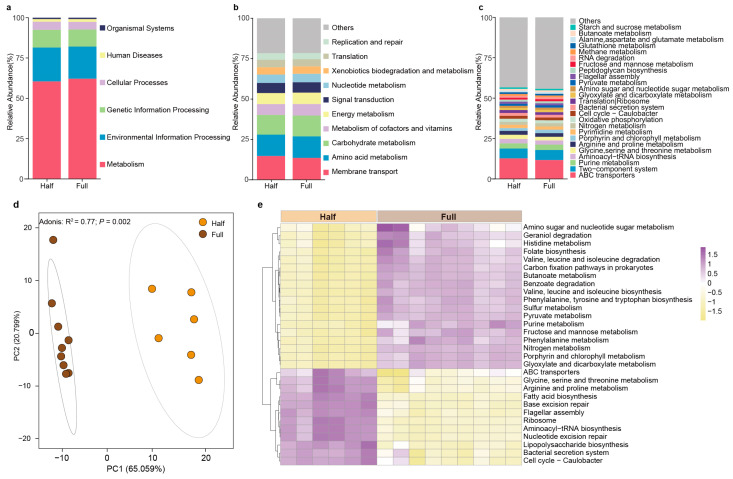
Comparison of antler velvet microbial function between the Half and Full groups. The relative abundances of metabolic pathways at KEGG level 1 (**a**), KEGG level 2 (**b**), and KEGG level 3 (**c**); (**d**) PCoA revealing variations in KEGG level 3 between the Half and Full groups; and (**e**) the significantly changed pathways based on LEfSe analysis at level 3 (LDA score [log 10] > 2.5, *p* < 0.05).

We used Tax4Fun to predict the potential functions of the antler velvet microbiota. The results revealed six pathways in both the Half and Full groups at KEGG level 1, including metabolism, environmental information processing, genetic information processing, cellular processes, human diseases, and organ systems, with metabolism being the most abundant pathway (Figure 4a). At KEGG level 2, the primary predicted pathways included membrane transport, amino acid metabolism, carbohydrate metabolism, metabolism of cofactors and vitamins, energy metabolism, signal transduction, nucleotide metabolism, xenobiotics biodegradation and metabolism, translation, and replication and repair (Figure 4b). At KEGG level 3, the primary predicted pathways included ABC transporters, two-component system, purine metabolism, aminoacyl-tRNA biosynthesis, glycine, serine, and threonine metabolism, arginine and proline metabolism, porphyrin and chlorophyll metabolism, pyrimidine metabolism, nitrogen metabolism, and oxidative phosphorylation (Figure 4c).

We then analyzed the differences in the functions of the antler velvet microbiota between the Half and Full groups at KEGG level 3. The results show that there were distinct differences in microbial functional potential between the Half group and the Full group (Figure 4d, *p* < 0.05). We found that 30 pathways were significantly different between the Half and Full groups at KEGG level 3, including purine metabolism, geraniol degradation, porphyrin and chlorophyll metabolism, folate biosynthesis, fatty acid biosynthesis, lipopolysaccharide biosynthesis, sulfur metabolism, nitrogen metabolism, pyruvate metabolism, glyoxylate and dicarboxylate metabolism, fructose and mannose metabolism, butanoate metabolism, amino sugar and nucleotide sugar metabolism, valine, leucine and isoleucine degradation, valine, leucine and isoleucine biosynthesis, phenylalanine, tyrosine and tryptophan biosynthesis, phenylalanine metabolism, histidine metabolism, glycine, serine, and threonine metabolism, arginine and proline metabolism, ribosome, bacterial secretion system, ABC transporters, and flagellar assembly (Figure 4e). Among them, the relative abundances of glycine, serine, and threonine metabolism, arginine and proline metabolism, fatty acid biosynthesis, and ABC transporters were significantly increased in the Half group, while five carbohydrate metabolism pathways (pyruvate metabolism, glyoxylate and dicarboxylate metabolism, fructose and mannose metabolism, butanoate metabolism and amino sugar, and nucleotide sugar metabolism) and five amino acid metabolism pathways (valine, leucine, and isoleucine degradation, valine, leucine, and isoleucine biosynthesis, phenylalanine, tyrosine, and tryptophan biosynthesis, phenylalanine metabolism, and histidine metabolism) were significantly increased in the Full group.

Correlation analyses were performed on the differential KEGG pathways and microbial composition at the genus level (Figure S2). The result showed that *Staphylococcus* spp., *Romboutsia*_B, *Clostridium*_T, *Dietzia* spp., *Clostridioides*_A, *Brevibacillus*_D, and *Fastidiosipila* spp. positively correlated with glycine, serine, and threonine metabolism, arginine and proline metabolism, fatty acid biosynthesis, and ABC transporters (*p* < 0.05), while *Atopostipes* spp., *Faecousia* spp., *Albibacterium* spp., *Cryptobacteroides* spp., *Psychrobacter* spp., *Ornithinimicrobium*_390112, *Oceanobacillus* spp., *Prevotella* spp., *Pontibacter*_909912, and *Fibrobacter* spp. positively correlated with five carbohydrate metabolism pathways (pyruvate metabolism, glyoxylate and dicarboxylate metabolism, fructose and mannose metabolism, butanoate metabolism and amino sugar, and nucleotide sugar metabolism) and five amino acid metabolism pathways (valine, leucine, and isoleucine degradation, valine, leucine, and isoleucine biosynthesis, phenylalanine, tyrosine, and tryptophan biosynthesis, phenylalanine metabolism, and histidine metabolism) (*p* < 0.05).

## 4. Discussion

In this study, we examined the microbial composition and dynamics of antler velvet during regeneration using 16S rRNA gene sequencing. The prevalent bacterial phyla of antler velvet were Firmicutes, Proteobacteria, Actinobacteriota, and Bacteroidota, which is consistent with the findings of microbial composition on the skin of humans [39], mice [40], dogs [16], pigs [41], sheep [19], and yaks and cattle [42]. We found that the dominant bacterial genera in antler velvet were *Staphylococcus* spp., *Atopostipes* spp., *Corynebacterium* spp., and *Burkholderia* spp., which is slightly different from previous findings from the skin of other mammals, such as the dominated genera of human skin (*Propionibacterium* spp., *Corynebacterium* spp., and *Staphylococcus* spp.) [39], the udder skin of dairy cows (*Corynebacterium* spp., *Bifidobacterium* spp., *Staphylococcus* spp., and *Brachybacterium* spp.) [43], and the dorsal and abdominal skin of donkeys (*Staphylococcus* spp., *Planococcus* spp., *Corynebacterium* spp., and *Salinicoccus* spp.) [44]. This difference is likely related to the living environment (lifestyle, hygiene routine, geographic location, climate, and seasonality) and/or skin physiology (moist, dry, and sebaceous microenvironments) [45,46]. However, the genera *Staphylococcus* spp. and *Corynebacterium* spp. were commonly present on the skin of these mammals, suggesting that these bacteria may have a core symbiotic relationship with skin.

We also observed a significant decrease in the microbial diversity of antler velvet in the Half group compared with the Full group. Similar to our findings, previous studies have demonstrated that the microbial diversity of human skin in chronic wounds [47] and burn wounds [48] is also significantly lower than that of healthy skin. Sanjar et al. [49] reported that the microbial diversity of rat burn wounds was lower than that of healthy skin. Similarly, the microbial diversity observed in horse limb and thoracic wounds during the early healing stages (1 week and 2 weeks post-wound) is lower than that observed during the fully healed stage [50]. A decrease in microbial diversity and richness is often associated with skin diseases and inflammation such as psoriasis [51], atopic dermatitis [52], and vitiligo [53]. Furthermore, Gupta et al. (2005) found that *Pseudomonas* spp. in burn wounds significantly inhibited growth of the opportunistic fungus *Candida* spp. [54]. It was reported that antler velvet regeneration is associated with an attenuated immune response [8]. These results indicate that the immune response may be an important factor influencing the microbial composition of antler velvet.

Compared to the Full group, the relative abundances of *Staphylococcus* spp., *Romboutsia*_B, and *Dietzia* spp. increased significantly in the Half group, while *Atopostipes* spp., *Psychrobacter* spp., and *Faecousia* spp. increased in the Full group. Similar to our results, Sanjar et al. [49] found that the proportion of *Staphylococcus* spp. in rat burn wounds was significantly higher than that in sham burns. Hannigan et al. (2014) demonstrated that the relative abundance of *Staphylococcus* spp. in the center of open fracture wounds increased significantly with the course of injury [55]. The genus *Romboutsia* has multiple metabolic capacities, such as anaerobic respiration, single amino acid fermentation, and the production of metabolic end products [56]. Recent studies showed that *Romboutsia* spp. were drastically reduced in adenomatous polyps and cancerous mucosa [57]. *Dietzia* spp. can colonize human skin and are enriched in lipid metabolism [58,59]. Moreover, the microbiota correlations in the Half group were much more complex than those in the Full group. These results suggest that the cooperation of different microbiota likely affects velvet regeneration. *Staphylococcus* spp. is a Gram-positive bacterium that inhabits the skin and mucous membranes [60] and has been reported to be negatively correlated with ulcer depth and duration [61] and burn infection [62]. *S. epidermidis* stimulates skin CD8^+^ T cells, which enhance keratinocyte progression by upregulating Toll-like receptors and modulating TNF-α signaling [63,64]. Additionally, lipoteichoic acid [15] and trace amines [65] produced by *S. epidermidis* can act upon keratinocytes to inhibit skin inflammation or antagonize the inhibition of cell migration to accelerate wound healing. However, the growth of *S. aureus* is associated with severe skin infections, primarily due to the production of superantigens [66,67]. These results suggest functional heterogeneity within *Staphylococcus* spp. Further investigation is warranted to isolate *Staphylococcus* species and examine their genomic profiles.

The relative abundances of glycine, serine, and threonine metabolism, arginine and proline metabolism were significantly increased in the Half group in comparison with the Full group, and were also positively correlated with *Staphylococcus* spp., *Romboutsia_B* and *Dietzia* spp. Metabolism of nutrients such as amino acids and lipids promotes skin wound healing and reduces scar formation by providing energy and substrate for repair cells [68]. Amino acids are important nutrients required for wound healing promotion and repair of the damaged skin [69]. Arginine can be hydrolyzed to ornithine, which is further metabolized to proline and polyamine. Proline is an important component of collagen, and polyamine is important for cell proliferation and differentiation [70,71]. Previous findings have also shown that the addition of amino acid precursors of collagen (proline, lysine, glycine, and leucine) can promote and accelerate wound healing [72]. These findings suggest that interactions among *Staphylococcus* spp., *Romboutsia* spp., and *Dietzia* spp., and amino acids (such as arginine and proline) can affect wound healing and regeneration of antler velvet. *Atopostipes* spp. are lactic acid bacteria that can metabolize glucose to formate, acetate, and lactate, and valine to branched-chain fatty acids, which are abundant in the skin and can inhibit the growth of potential pathogens [73,74,75,76]. *Psychrobacter* spp. are prevalent on the skin of humpback whales, and changes in their prevalence seem to be related to changes in the animal’s metabolic state [77]. *Faecousia* spp. are often detected in animal feces and are related to carbohydrate degradation [78]. Consistently, the metabolism of carbohydrates was also enhanced in the Full group, suggesting that these microbes might influence the carbohydrate metabolism of antler velvet during the later stages of regeneration. These results indicate possible shifts in the metabolic profiles of the antler velvet microbiota, highlighting the role of the interaction between specific microbiota and amino acids in antler velvet regeneration.

## 5. Conclusions

Here, we characterized and compared the bacterial communities that are present during the regeneration of antler velvet. Although common microbiota components were observed between antler velvet and the skin of other mammals, antler velvet was also inhabited by a slightly distinct microbiota. The immune response is likely an important factor influencing the dynamics of the antler velvet microbiota. The significantly changed microbial diversity, composition, and potential functions suggest a role for *Staphylococcus* and arginine and proline metabolism during antler velvet regeneration. Our results expand the current understanding of the link between microbiota and skin regeneration.

## Data Availability

The sequences in the present study were deposited in the SRA database under accession number SRP520253.

## Supplementary Materials

The following supporting information can be downloaded at: https://www.mdpi.com/article/10.3390/microorganisms13010036/s1, Table S1. Summarizes data regarding the OTUs and microbial phylum, family, and genus levels. Figure S1. The Half (a) and Full (b) regeneration periods of antler velvet. Figure S2. Correlation analysis among differential pathways and microbial genera.
