# Supplementary figures and images for: Characteristics and Differences in the Antler Velvet Microbiota During Regeneration
Source: Microorganisms. 2024 Dec 27;13(1):36. doi: 10.3390/microorganisms13010036 (PMC11768019; doi:10.3390/microorganisms13010036)

**a**

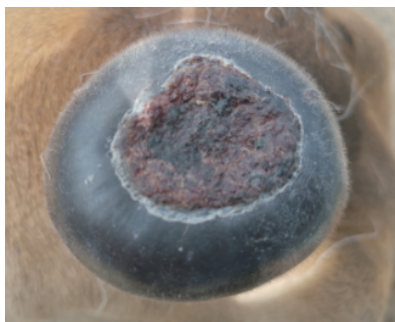

**b**

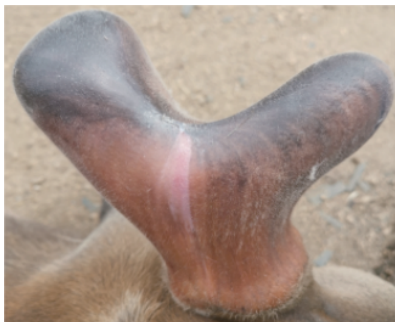

Supplement: Supplementary file 1 [file microorganisms-13-00036-s001.zip › Supplementary figure S1.pdf]

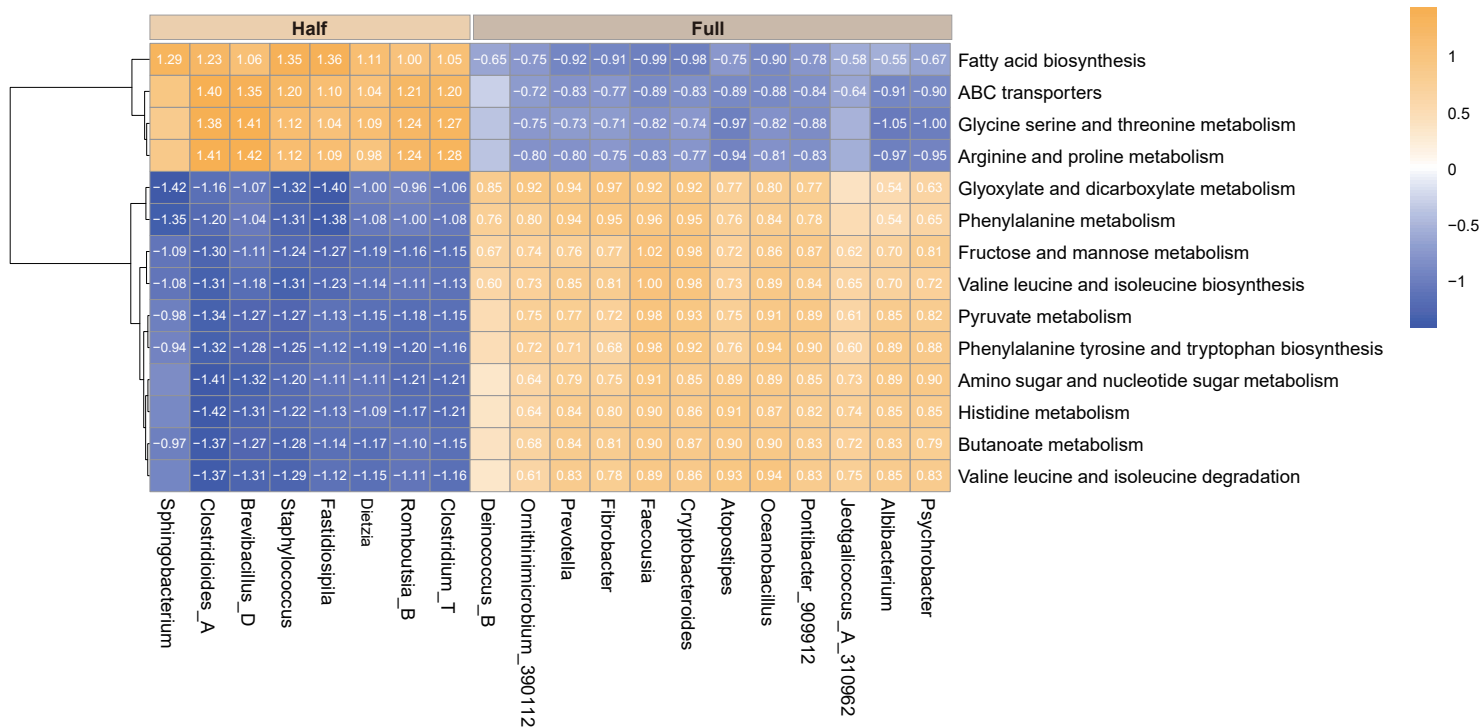

Supplement: Supplementary file 1 [file microorganisms-13-00036-s001.zip › Supplementary figure S2.pdf]
